# Calcium Transport Proteins in Fungi: The Phylogenetic Diversity of Their Relevance for Growth, Virulence, and Stress Resistance

**DOI:** 10.3389/fmicb.2019.03100

**Published:** 2020-01-28

**Authors:** Mario Lange, Edgar Peiter

**Affiliations:** Plant Nutrition Laboratory, Institute of Agricultural and Nutritional Sciences, Faculty of Natural Sciences III, Martin Luther University of Halle-Wittenberg, Halle (Saale), Germany

**Keywords:** calcium signal, calcium signaling, calcium channel, calcium pump, calcium proton antiporter, filamentous fungi, yeast

## Abstract

The key players of calcium (Ca^2+^) homeostasis and Ca^2+^ signal generation, which are Ca^2+^ channels, Ca^2+^/H^+^ antiporters, and Ca^2+^-ATPases, are present in all fungi. Their coordinated action maintains a low Ca^2+^ baseline, allows a fast increase in free Ca^2+^ concentration upon a stimulus, and terminates this Ca^2+^ elevation by an exponential decrease – hence forming a Ca^2+^ signal. In this respect, the Ca^2+^ signaling machinery is conserved in different fungi. However, does the similarity of the genetic inventory that shapes the Ca^2+^ peak imply that if “you’ve seen one, you’ve seen them all” in terms of physiological relevance? Individual studies have focused mostly on a single species, and mechanisms elucidated in few model organisms are usually extrapolated to other species. This mini-review focuses on the physiological relevance of the machinery that maintains Ca^2+^ homeostasis for growth, virulence, and stress responses. It reveals common and divergent functions of homologous proteins in different fungal species. In conclusion, for the physiological role of these Ca^2+^ transport proteins, “seen one,” in many cases, does not mean: “seen them all.”

## Introduction

**FIGURE 1:**
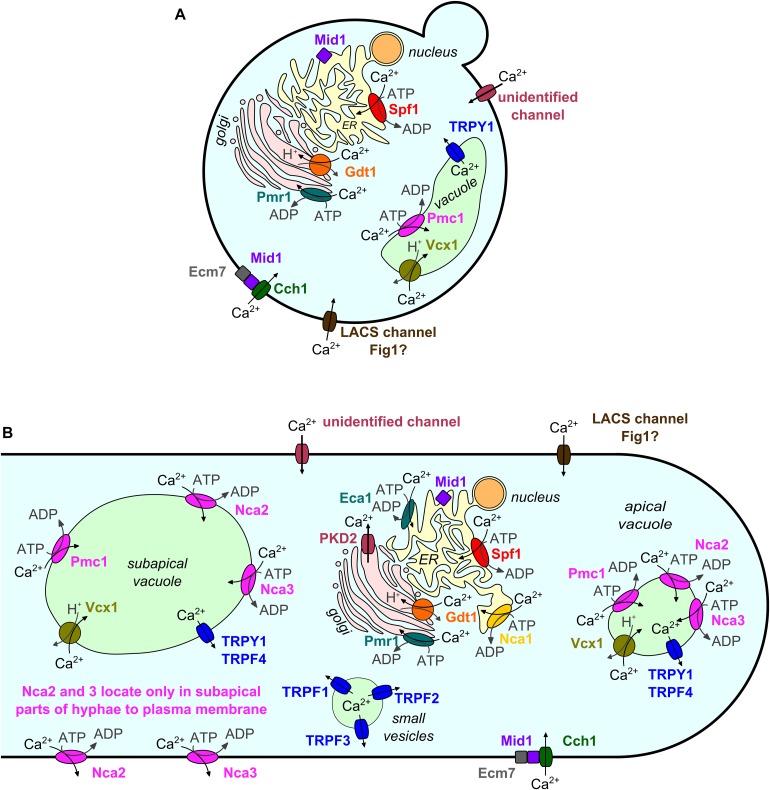
Subcellular localization of Ca^2+^ channels, Ca^2+^/H^+^ exchangers, and Ca^2+^ ATPases in the model yeast *S. cerevisiae* **(A)** and other fungi **(B)**. Homologs are depicted in identical colors. Subcellular localizations are shown as described or assumed in the literature. Data from fungi other than *S. cerevisiae* are depicted in a simplified model of a fungal hypha as most of these data were gained from filamentous fungi. Note the more complex localization patterns and larger number of protein family members in non-yeast fungi.

In fungi, as in other higher organisms, many stimuli and developmental cues excite calcium (Ca^2+^) signals, which again initiate appropriate downstream responses by changing the conformation of Ca^2+^-binding proteins. Ca^2+^ signals are usually characterized by a sharp rise in cytosolic free Ca^2+^ ([Ca^2+^]_cyt_) followed by an exponential decrease (Cui et al., 2009; Carbó et al., 2017). The basal [Ca^2+^]_cyt_ level is low (∼100 nM in *N*eurospora *crassa*; Tamuli et al., 2013). This level is maintained by Ca^2+^ pumps and antiporters that export Ca^2+^ or sequester it into organelles (in *N. crassa* mainly into the vacuole; Tamuli et al., 2013). In response to a stimulus, Ca^2+^ channels open and allow Ca^2+^ to passively enter the cytosol along the concentration gradient from extracellular space or intracellular stores. Ca^2+^-sensitive Ca^2+^ channels may further amplify the signal by Ca^2+^-induced Ca^2+^ release (CICR) (Goncalves et al., 2014). Ca^2+^/H^+^ antiporters utilize the proton motive force, and Ca^2+^ pumps use ATP to transport Ca^2+^ against a concentration gradient out of the cytosol. Thereby, Ca^2+^/H^+^ antiporters and Ca^2+^ pumps decrease the [Ca^2+^]_cyt_ again to the basal level. This set of Ca^2+^ transport proteins identified in the model yeast *Saccharomyces cerevisiae* is displayed in Figure 1A, and equivalent mechanisms found in other fungi are shown in Figure 1B. For details on mechanisms of Ca^2+^ homeostasis, the reader is referred to excellent general reviews, for example Cunningham (2011) for yeast or Tamuli et al. (2013) for *N. crassa*. A simulation of Ca^2+^ homeostasis in yeast is presented by Cui et al. (2009). Supplementary File 1 contains a collection of recent studies on the regulation of Ca^2+^ transport and homeostasis, which is not the focus of this mini-review.

As in other organisms, in fungi Ca^2+^ signals are decoded and modulated by Ca^2+^-sensitive proteins, such as calmodulin (CaM) and calcineurin (CN). CaM binds to CaM-dependent proteins and modulates their activity by Ca^2+^-induced conformational changes. The protein phosphatase CN is activated by Ca^2+^ itself and by CaM. CN activates the transcription factor Crz1 by dephosphorylation, thus triggering its translocation into the nucleus (Stathopoulos-Gerontides et al., 1999). Crz1 is also a central downstream target of Ca^2+^ signals in filamentous fungi (Schumacher et al., 2008; Choi et al., 2009).

A considerable number of studies have elucidated the processes that contribute to the generation of Ca^2+^ signals and the physiological roles of Ca^2+^ transport proteins in fungi. Thereby, individual studies focus mostly on a single species, and mechanisms elucidated in few model species are usually extrapolated to other species. In this mini-review, we query the validity of this generalization by comparing findings on the impact of the Ca^2+^ signaling machinery in diverse fungal species. The phylogenetic diversity of mutant phenotypes with respect to growth, branching, surface recognition, sporulation, and virulence, as well as resistance to diverse stresses is condensed in Table 1. Throughout this review, the phenotype descriptions refer to this table.

**TABLE 1:** Compilation of observed phenotypes for Ca^2+^-signaling defect mutants in different fungi. For explanation of colors and symbols, see legend below table.

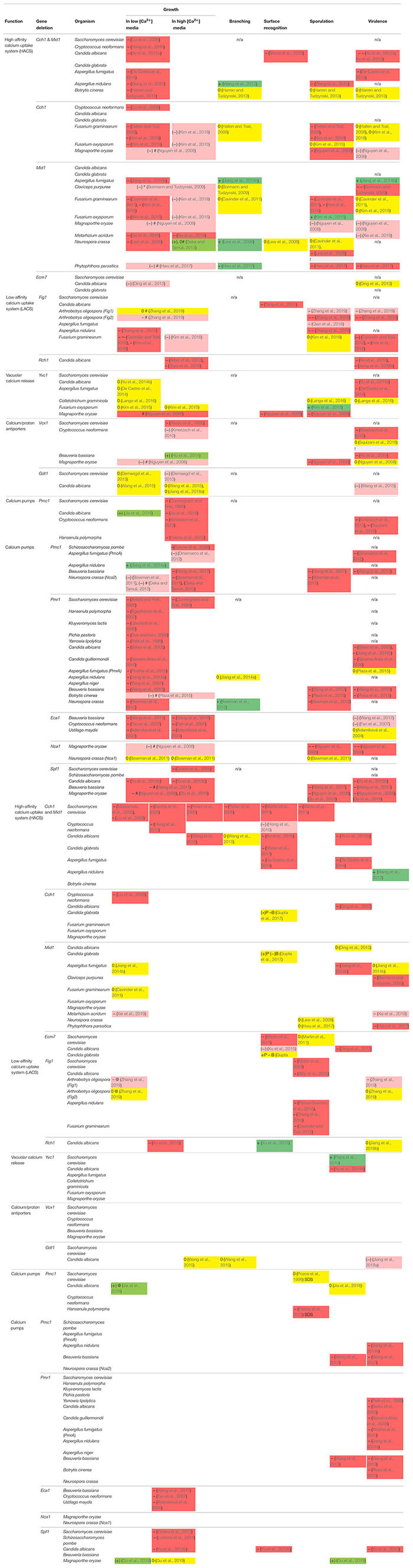

## Ca^2+^ Channels – Generating a Ca^2+^ Signal Upon a Stimulus

### The High-Affinity Ca^2+^ Uptake System in the Plasma Membrane

In *S. cerevisiae*, a high-affinity Ca^2+^ uptake system (HACS) is formed by Cch1, Mid1, and Ecm7. Cch1 is a homolog of the α subunit of mammalian L-type voltage-gated Ca^2+^ channels (Fischer et al., 1997). The transmembrane protein Mid1 interacts with Cch1 (Locke et al., 2000). Consistently, most phenotypes of deletions in either one or both genes are identical. However, MID1 has also been claimed to function independently as stretch-activated Ca^2+^-permeable channel localized largely in the ER (Kanzaki et al., 1999; Yoshimura et al., 2004). Ecm7 is involved in HACS-mediated Ca^2+^ influx, but deletion phenotypes are less drastic than those of Δ*cch1* or Δ*mid1* mutants (Martin et al., 2011; Kato et al., 2017).

In good agreement with the high affinity of HACS for Ca^2+^, the system is important for growth of diverse fungal species when external Ca^2+^ is limited. *Claviceps purpurea* is a notable exception in that a Δ*mid1* strain grows less vigorously than the wild type, but addition of Ca^2+^ inhibits growth even further. However, in *N. crassa* high-Ca^2+^ media lead to enhanced growth (Deka and Tamuli, 2013). In *Fusarium graminearum*, growth was more strongly affected in *mid1* than in *cch1* mutants. Only *mid1* of *F. graminearum* produced more conidia, again pointing to independent functions of this HACS subunit (Kim et al., 2015). In some fungal species, reduced growth upon HACS deletion is associated with hyperbranching or a defect in surface recognition, while for others this is not the case. Sporulation and the tolerance to a wide variety of stresses depend on HACS in many fungi, but this requirement varies to some extent between species. The importance of HACS for virulence strongly depends on the fungal species, and ranges from essential to detrimental. In summary, the role of HACS for growth in low [Ca^2+^] environments is widely conserved, but other functions of the HACS vary more or less between different fungi.

### The Low-Affinity Ca^2+^ Uptake System in the Plasma Membrane

The molecular identity of the low-affinity Ca^2+^ uptake system (LACS) is still unclear. Fig1, a plasma membrane protein, has been proposed to be either the LACS Ca^2+^ channel itself or an important regulator of it. It is needed for Ca^2+^ influx and normal mating in all fungi analyzed so far. In *S. cerevisiae* and *Candida albicans*, Fig1 was shown to be important for Ca^2+^ influx during mating and cell fusion (Muller et al., 2003; Yang et al., 2011). Deletion of *Fig1* also causes retardation in vegetative growth, which can be rescued by the addition of Ca^2+^ in *N. crassa* but not in *F. graminearum*. In the latter species, Fig1 is more important than HACS in the generation of disease symptoms (Kim et al., 2018). *Arthrobotrys oligospora* has two *Fig* genes. Here, Fig1 is more important for stress tolerance, while Fig2 is crucial for growth, sporulation, and virulence (Zhang et al., 2019).

The plasma membrane protein Rch1 is a negative regulator of Ca^2+^ uptake, but the underlying mechanism is not clear (Alber et al., 2013). *Rch1* is expressed under high-Ca^2+^ stress and important for growth in these conditions (Zhao et al., 2016). In *C. albicans*, Rch1 is essential for full virulence, whereby it genetically interacts with CaPMR1 (Jiang et al., 2018b).

### Ca^2+^ Release Channels in the Vacuolar Membrane

TRPY1 (synonym Yvc1) is a Ca^2+^ channel of the Transient Receptor Potential family in the vacuolar membrane of *S. cerevisiae* (Palmer et al., 2001; Denis and Cyert, 2002; Hamamoto et al., 2018; Amini et al., 2019). It is activated by stretch (Zhou et al., 2003) and amplifies hyperosmotic shock-triggered Ca^2+^ signals by CICR (Su et al., 2009). In these aspects, TRPY1s of *Kluyveromyces lactis* (Zhou et al., 2005), *C. albicans* (Zhou et al., 2005), and the filamentous fungus *F. graminearum* (Ihara et al., 2013) resemble ScTRPY1. However, the channel of *F. graminearum*, but not that of *S. cerevisiae*, is negatively regulated by inositol phosphates (Ihara et al., 2013). The physiological relevance of TRPY1 is highly diverse between different fungi. In *S. cerevisiae*, a deletion of TRPY1 leads to increased resistance to oxidative stress (Popa et al., 2010); no other phenotypical differences were reported (Chang et al., 2010). In contrast, *C. albicans* needs TRPY1 to survive oxidative stress (Yu et al., 2014b). In *C. albicans* (Yu et al., 2014a) and *Aspergillus fumigatus* (De Castro et al., 2014), TRPY1 is important for biofilm formation and virulence, but not for growth on agar. *Colletotrichum graminicola* has four TRPY1 homologs (Lange et al., 2016). In this fungus, deletion of any of those genes did not lead to any differences in Ca^2+^ signal generation, in growth with or without stress, or in virulence. In contrast to all other fungal species analyzed so far, *Magnaporthe oryzae* requires TRPY1 for axenic growth on agar and for virulence (Nguyen et al., 2008). In summary, the physiological roles of TPRY1 homologs are highly diverse in the fungi studied so far.

## Ca^2+^/H^+^ Antiporters – High-Capacity Low-Affinity Ca^2+^ Sequestration

### Ca^2+^/H^+^ Exchange Over the Vacuolar Membrane

Vcx1 is a low-affinity, high-capacity Ca^2+^/H^+^ antiporter in the vacuolar membrane of *S. cerevisiae.* It can also sequester Mn^2+^. Therefore, Vcx1 allows cells to grow at very high extracellular concentrations of these ions (Cunningham and Fink, 1996; Pozos et al., 1996). Vcx1 serves to recover basal [Ca^2+^]_cyt_ after a Ca^2+^ peak in *S. cerevisiae*, whereas the vacuolar Ca^2+^ ATPase Pmc1 (see the section “Ca^2+^-ATPases Sequestering Ca^2+^ Into the Vacuole and Exporting Ca^2+^ out of the Cell”) maintains the basal [Ca^2+^]_cyt_ prior to a signal (Denis and Cyert, 2002). This recovery is slowed down by a repression of Vcx1 by Ca^2+^-activated CN (Rusnak and Mertz, 2000).

Vcx1 of *Cryptococcus neoformans* also localizes to the vacuolar membrane and is needed to grow on high Ca^2+^ but not on standard media (Kmetzsch et al., 2010). Here, both the antiporter and the ATPase maintain the [Ca^2+^]_cyt_ baseline and sequestrate cytosolic Ca^2+^ after a peak (Kmetzsch et al., 2013). The effect of *vcx1*Δ for virulence of this fungus is disputed (Kmetzsch et al., 2010; Squizani et al., 2018). A knockdown of each of the four *M. oryzae Vcx1* genes results in no to slight reduction of growth speed on standard media. It causes a clear reduction in sporulation and appressorium formation, but there is no effect on pathogenicity (Nguyen et al., 2008). In contrast, deletion of individual *Vcx1* genes in the insect-pathogenic fungus *Beauveria bassiana*, which has five *Vcx1* homologs, results in a moderate reduction in pathogenicity. In this fungus growth is not impaired by *Vcx1* deletion on standard media, while there is a slight effect on high-Ca^2+^ media (Hu et al., 2014).

In summary, sequestering high Ca^2+^ concentrations seems to be the common job of Vcx1, while effects on growth and virulence are highly species-specific.

### Ca^2+^/H^+^ Exchange Over Golgi Membranes

Gdt1 is a putative Ca^2+^/H^+^ and Mn^2+^/H^+^ antiporter of *S. cerevisiae* which is localized to membranes of the *cis*- and *medial*-Golgi. It is believed to be important for supplying the Golgi with Ca^2+^ and Mn^2+^, and for sequestration of high [Ca^2+^]_cyt_ (Demaegd et al., 2013; Colinet et al., 2016). *Gdt1* deletion causes late-Golgi glycosylation defects in particular in high-Ca^2+^ media, pointing to a primary role in Mn^2+^ transport for glycosylation (Dulary et al., 2018). Consensus motives in the transmembrane helices 1 and 4 are important for Gdt1 function (Colinet et al., 2017).

A Gdt1 homolog of *C. albicans* complements the respective deletion in *S. cerevisiae* (Wang et al., 2015). Gdt1 has been suggested to remove Ca^2+^ from the cytosol also in this fungus (Jiang et al., 2018a), and the mutant shows a reduced virulence (Wang et al., 2015). There is clearly more research required on the function and the physiological roles of the Gdt1 family in different fungi.

## Ca^2+^-ATPases – Keeping the Cytosolic Free Ca^2+^ Concentration at a Low Basal Level and Supplying Organelles With Ca^2+^

### Ca^2+^-ATPases Sequestering Ca^2+^ Into the Vacuole and Exporting Ca^2+^ Out of the Cell

The Ca^2+^-ATPase Pmc1 localizes to the vacuolar membrane and mediates Ca^2+^ sequestration, which is essential for growth of *S. cerevisiae* and *C. albicans* in high-Ca^2+^ media. Pmc1 activity is partially inhibited through physical interaction with Nyv1 at basal [Ca^2+^]_cyt_ (Takita et al., 2001). Under conditions of high [Ca^2+^]_cyt_, expression of *Nyv1* stays constant, while *Pmc1* expression is induced via CaM-CN-Crz1 signaling to keep [Ca^2+^]_cyt_ stable.

*Aspergillus fumigatus* harbors three *Pmc1* homologs (*PmcA*, *PmcB*, and *PmcC*). A deletion of *PmcC* is lethal. Deletion of *PmcA* results in impairment of spore germination, growth at high [Ca^2+^] in rich (but not in minimal) media, and virulence (Dinamarco et al., 2012). *B. bassiana* also has three *Pmc* genes. Here, Δ*pmcB* is massively impaired in growth, while Δ*pmcC* is vital and only slightly impaired, and Δ*pmcA* grows like the wild type. *PmcA-C* of *B. bassiana* are important for full germination speed, conidiation, resistance to oxidative and cell wall stress, as well as for virulence (Wang et al., 2017). Pmc1 of *C. neoformans* is needed for growth on Ca^2+^-supplemented rich media, replication in its host, and virulence (Kmetzsch et al., 2013; Squizani et al., 2018). The non-pathogenic filamentous fungus *N. crassa* has two Pmc-type Ca^2+^-ATPases, Nca2 and Nca3 (for Nca1 see the section “Ca^2+^-ATPases Sequestering Ca^2+^ and Mn^2+^ Into the Golgi and ER”). Both locate to the vacuolar membrane network (VMN) and subapically also to the plasma membrane, as their mammalian homolog. Nca2 is needed to supply the VMN and export Ca^2+^ out of the cell. The protein is beneficial for growth in minimal media with low [Ca^2+^] and essential when [Ca^2+^]_ext_ is high. Female spores of *nca2*Δ strains are infertile, and both genders produce less spores. Nca3 seems to be dispensable for growth, sporulation, and stress tolerance (Bowman et al., 2011).

Pmc1 sequesters Ca^2+^ into the vacuole in all fungi analyzed so far. In yeasts, Pmc1 is dispensable for normal growth but important under stressful conditions, whereas in other fungi this protein is important during the normal life cycle. In *A. fumigatus* and *C. neoformans* mutants for *Pmc1* homologs, the impact of high [Ca^2+^]_ext_ stress is greatly increased in richer media. Moreover, organisms with several *Pmc* genes show a considerable diversity in their relative importance.

### Ca^2+^-ATPases Sequestering Ca^2+^ and Mn^2+^ Into the Golgi and ER

The phylogeny of another group of Ca^2+^-ATPases is separated in two clades: The Golgi-localized Pmr1 branch and the ER-borne SERCA-type Eca1/Nca1 branch (Antebi and Fink, 1992; Adamíková et al., 2004). Some fungi have only genes belonging to one of these types in their genome, but this is not conserved (Fan et al., 2007; Wang et al., 2017).

Pmr1 is required for growth in many yeast fungi, especially in low-Ca^2+^ or low-Mn^2+^ media. In *S. cerevisiae*, it also supports vitality of stationary phase cells irrespective of medium [Ca^2+^] (Rudolph et al., 1989). It is essential for protein mannosylation in different yeast species (Antebi and Fink, 1992; Bates et al., 2005; Agaphonov et al., 2007; Navarro-Arias et al., 2016). *Pmr1*Δ strains of *C. albicans* and *Candida guilliermondii* show a massively reduced virulence next to the phenotypes mentioned above (Bates et al., 2005; Navarro-Arias et al., 2016). In contrast to *S. cerevisiae*, reduced vitality is recovered by high external [Ca^2+^] in *C. albicans* (Bates et al., 2005).

*Pmr1* is also important for growth of filamentous fungi, and even more so when Ca^2+^ availability is limited. These growth defects can be rescued in *B. bassiana* and *N. crassa* by addition of Mn^2+^ or Ca^2+^ (Bowman et al., 2012; Wang et al., 2013). Interestingly, growth defects in *A. fumigatus* and *Aspergillus nidulans* can be recovered by osmotic stabilization, but not by Ca^2+^ or Mn^2+^ (Pinchai et al., 2010; Jiang et al., 2014a). As in yeasts, *Pmr1* is needed in filamentous fungi to resist cell wall stress. The relevance of *Pmr1* for virulence of pathogenic fungi ranges from minor to highly important.

The *Eca1* branch of this Ca^2+^-ATPase family was first revealed in *Ustilago maydis*. Eca1 resides, similar to mammalian SERCAs, in the ER (Adamíková et al., 2004). It was also found in *C. neoformans* (Fan et al., 2007). In *B. bassiana* both *Pmr1* and *Eca1* are present (Wang et al., 2017). In these fungi, Eca1 is responsible for removing excessive Ca^2+^ from the cytosol. Furthermore, it supplies the ER lumen with essential Ca^2+^ (Adamíková et al., 2004; Fan et al., 2007; Wang et al., 2017). *Eca1* is important for growth, tolerance of ER stress, and Ca^2+^ signaling (Adamíková et al., 2004; Fan et al., 2007). The impact on virulence ranges from absent in *U. maydis*, via host- and temperature-dependent impairment in *C. neoformans*, to attenuated in *B. bassiana*.

Nca1 is closely related to Eca1. In *N. crassa*, Nca1 locates to the ER. In this fungus, deletion of *Nca1* causes no phenotype (Bowman et al., 2011). However, in *M. oryzae*, knockdown of *Nca1* results in a complete blockage of sporulation – rendering the fungus apathogenic – and in a slight reduction of colony growth (Nguyen et al., 2008).

In summary, there are only subtle functional differences within the Pmr1 branch between yeast and filamentous fungi. In spite of the bipartite phylogenetic relationship of the Pmr1 and SERCA-type ATPase family members, their molecular functions are quite similar. However, their relevance for virulence differs greatly between fungal species.

### An Emerging Family of ER-Localized Ca^2+^-ATPases

Another Ca^2+^/Mn^2+^-ATPase, Spf1, is also localized to the ER membrane of *S. cerevisiae*. It supplies the ER lumen with Ca^2+^/Mn^2+^ and removes excessive cytosolic amounts of these ions (Suzuki and Shimma, 1999; Cronin et al., 2000; Cohen et al., 2013). Deletion of *Spf1* results in reduced growth on high-Ca^2+^ media (Cronin et al., 2000) and defective sterol homeostasis (Sørensen et al., 2019). Furtheron, Δ*Spf1* leads to Ca^2+^/Mn^2+^ deficiency in the ER lumen, which provokes protein misfolding and reduced resistance to ER stress (Cronin et al., 2000; Cohen et al., 2013). The *Spf1* homolog of *Schizosaccharomyces pombe*, *Cta4*, has similar molecular functions as ScSpf1 (Lustoza et al., 2011). In *C. albicans*, *Spf1* deletion results in similar defects and represses the formation of the more pathogenic hyphal state (Yu et al., 2012b, 2013). *Spf1* of *M. oryzae* and *B. bassiana* is important for colony growth, sporulation, spore germination, and virulence (Nguyen et al., 2008; Wang et al., 2017; Qu et al., 2019). In most fungi, Spf1 is also important for stress tolerance.

Albeit examined in only few species yet, the function of Spf1 is, as known so far, very similar in different fungi as well as compared to the Pmr1/Eca1 family. This is contrasted by the clearly more diverse functions of Pmc1 in different species (see the section “Ca^2+^-ATPases Sequestering Ca^2+^ Into the Vacuole and Exporting Ca^2+^ Out of the Cell”). Therefore, phylogenetic similarity does not necessarily correlate with biological function.

## Conclusion and Outlook

A wealth of data on transport proteins that maintain Ca^2+^ homeostasis and shape Ca^2+^ signals in fungi has been acquired in the past. However, there is also evidence that we are still missing some fundamental parts of the fungal Ca^2+^ signalosome. First, pharmacological Ca^2+^ signaling modulators have pronounced effects on Ca^2+^ signals in fungi (Lange and Peiter, 2016), although the canonical targets of these chemicals are often not present in the respective species. Also, mathematical modeling (Cui et al., 2009) and experimental evidence (Goncalves et al., 2014) indicate that at least two additional Ca^2+^ channels (next to Cch1-Mid1) must exist in the fungal plasma membrane. The LACS component Fig1 may be one interesting candidate in this respect.

Regarding the known players, the physiological role of some of the proteins involved in shaping Ca^2+^ signals (Pmr1, Eca1, and Spf1) appears to be quite conserved during fungal evolution, whereas in others (Mid1, TRPY1/Yvc1), there appear to be striking differences between species. Therefore, the “seen one, seen them all” principle should be applied very cautiously, in particular in translational studies aiming to develop antifungal drugs. An ensuing question remains to be answered however: What are the causes for this evolutionary divergence? Therefore, future work needs move from the description of phenotypes to the deciphering of mechanisms in phylogenetically diverse fungal species.

## Author Contributions

ML and EP conceived, drafted, and finalized the manuscript, approved the final version of the article, and agreed to be accountable for all aspects of the work.

## Conflict of Interest

The authors declare that the research was conducted in the absence of any commercial or financial relationships that could be construed as a potential conflict of interest.
