# Supplementary material for: Calcium Transport Proteins in Fungi: The Phylogenetic Diversity of Their Relevance for Growth, Virulence, and Stress Resistance
Source: Front Microbiol. 2020 Jan 28;10:3100. doi: 10.3389/fmicb.2019.03100 (PMC6997533; doi:10.3389/fmicb.2019.03100)
Supplement: Supplementary file 1 [file Data_Sheet_1.PDF]

Supplemental File 1

References on mechanisms of Ca^{2+} transport and homeostasis in fungi

Cch1, Mid1, Fig1

Carbó et al. (2017)
$[Ca^{2+}]_{cyt}$ bursts measured on single cell level in vegetative and mating pheromone-treated cells of *S. cerevisiae*, as correlated to Mid1, Fig1 and a so far unknown Ca^{2+} import system

Cho et al. (2017)
Interaction of Mid1 with Pma1 in *S. cerevisiae*; H^+ pumping activity of Pma1 may increase Ca^{2+} permeability of Cch1-Mid1

Gohain and Tamuli (2019)
Interaction of Mid1 with Ncs1, which is transcriptionally regulated by Crz1 in *Neurospora crassa*

Iida et al. (2017):
Relevance of the Mid1 N-terminal region for N-glycosylation and translocation to the plasma membrane and ER in *S. cerevisiae*

Kato et al. (2017)
Genetic analysis of the regulation of Cch1 by the γ subunit homolog Ecm7 and a novel Cch1 interactor, cortical ER protein Scs2, in *S. cerevisiae*

Mishra et al. (2017)
Relevance of Ca^{2+} signalling via Cch1/Mid1 in parallel to the Pkc1/Mpk1 pathway for perception and survival of mechanical stress in *S. cerevisiae*

Qian et al. (2018)
Fig1-dependent Ca^{2+} signals in *A. fumigatus* and *A. nidulans*

Wang et al. (2016)
Calcineurin A and CchA coordinate Ca^{2+} influx under salt stress in *A. nidulans*

TRPY1 (Yvc1)

Amini et al. (2019)
Patch clamp and Ca^{2+} imaging experiments identify inhibitory Ca^{2+}-binding sites in TRPY1 of *S. cerevisiae*

Hamamoto et al. (2018)
Electrophysiological characterization of *S. cerevisiae* TRPY1, demonstrating activation by reducing agents and regulation by inositol phosphates and Ca^{2+}

Peng et al. (2019)
TRPY1 determines polarized localization of the NADPH oxidase Fre8, which is important for hyphal tip-high reactive oxygen species gradient in germtubes

Wilson et al. (2018)
Interaction of PI(3,5)P2 and TRPY1 in the orchestration of vacuolar cation transport and osmoregulation in *S. cerevisiae*

Vcx1, Pmr1, Spf1
Kume et al. (2017)
Role of Vcx1 and Ca^{2+} signalling for G2 phase checkpoint passage in *S. pombe*

Sorensen et al. (2019)
Spf1 activity is stimulated by phosphatidylinositol 4-phosphate and maintains sterol homeostasis

Xu et al. (2019)
Cmk2 is a negative regulator of Ca^{2+}/calcineurin signalling and expression of *Pmr1* and *Pmc1* in *S. cerevisiae*

References

Amini, M., Wang, H., Belkacemi, A., Jung, M., Bertl, A., Schlenstedt, G., Flockerzi, V., and Beck, A. (2019). Identification of inhibitory Ca^{2+} binding sites in the upper vestibule of the yeast vacuolar TRP channel. *iScience* 11, 1-12. doi: 10.1016/j.isci.2018.11.037

Carbó, N., Tarkowski, N., Ipina, E.P., Dawson, S.P., and Aguilar, P.S. (2017). Sexual pheromone modulates the frequency of cytosolic Ca^{2+} bursts in *Saccharomyces cerevisiae*. *Mol. Biol. Cell* 28, 501-510. doi: 10.1091/mbc.E16-07-0481

Cho, T., Ishii-Kato, A., Fukata, Y., Nakayama, Y., Iida, K., Fukata, M., and Iida, H. (2017). Coupling of a voltage-gated Ca^{2+} channel homologue with a plasma membrane H^+-ATPase in yeast. *Genes Cells* 22, 94-104. doi: 10.1111/gtc.12458

Gohain, D., and Tamuli, R. (2019). Calcineurin responsive zinc-finger-1 binds to a unique promoter sequence to upregulate neuronal calcium sensor-1, whose interaction with MID-1 increases tolerance to calcium stress in *Neurospora crassa*. *Mol. Microbiol.* 111, 1510-1528. doi: 10.1111/mmi.14234

Hamamoto, S., Mori, Y., Yabe, I., and Uozumi, N. (2018). *In vitro* and *in vivo* characterization of modulation of the vacuolar cation channel TRPY1 from *Saccharomyces cerevisiae*. *FEBS J.* 285, 1146-1161. doi: 10.1111/febs.14399

Iida, K., Teng, J., Cho, T., Yoshikawa-Kimura, S., and Iida, H. (2017). Post-translational processing and membrane translocation of the yeast regulatory Mid1 subunit of the Cch1/VGCC/NALCN cation channel family. *J. Biol. Chem.* 292, 20570-20582. doi: 10.1074/jbc.M117.810283

Kato, T., Kubo, A., Nagayama, T., Kume, S., Tanaka, C., Nakayama, Y., Iida, K., and Iida, H. (2017). Genetic analysis of the regulation of the voltage-gated calcium channel homolog Cch1 by the g subunit homolog Ecm7 and cortical ER protein Scs2 in yeast. *PLOS ONE* 12, e0181436. doi: 10.1371/journal.pone.0181436

Kume, K., Hashimoto, T., Suzuki, M., Mizunuma, M., Toda, T., and Hirata, D. (2017). Identification of three signaling molecules required for calcineurin-dependent monopolar growth induced by the DNA replication checkpoint in fission yeast. *Biochem. Biophys. Res. Commun.* 491, 883-889. doi: 10.1016/j.bbrc.2017.07.129

Mishra, R., Van Drogen, F., Dechant, R., Oh, S., Jeon, N.L., Lee, S.S., and Peter, M. (2017). Protein kinase C and calcineurin cooperatively mediate cell survival under compressive mechanical stress. *Proc. Natl. Acad. Sci. USA* 114, 13471-13476. doi: 10.1073/pnas.1709079114

Peng, L., Yu, Q., Wei, H., Zhu, N., Ren, T., Liang, C., Xu, J., Tian, L., and Li, M. (2019). The TRP Ca^{2+} channel Yvc1 regulates hyphal reactive oxygen species gradient for maintenance of polarized growth in *Candida albicans*. *Fungal Genet. Biol.* 133, 103282. doi: 10.1016/j.fgb.2019.103282

Qian, H., Chen, Q., Zhang, S., and Lu, L. (2018). The claudin family protein FigA mediates Ca^{2+} homeostasis in response to extracellular stimuli in *Aspergillus nidulans* and *Aspergillus fumigatus*. *Front. Microbiol.* 9, 977. doi: 10.3389/fmicb.2018.00977

Sorensen, D.M., Holen, H.W., Pedersen, J.T., Martens, H.J., Silvestro, D., Stanchev, L.D., Costa, S.R., Gunther Pomorski, T., Lopez-Marques, R.L., and Palmgren, M. (2019). The P5A ATPase Spf1p is stimulated by phosphatidylinositol 4-phosphate and influences cellular sterol homeostasis. *Mol. Biol. Cell* 30, 1069-1084. doi: 10.1091/mbc.E18-06-0365

Wang, S., Liu, X., Qian, H., Zhang, S., and Lu, L. (2016). Calcineurin and calcium channel CchA coordinate the salt stress response by regulating cytoplasmic Ca^{2+} homeostasis in *Aspergillus nidulans*. *Applied and Environmental Microbiology* 82, 3420-3430. doi: 10.1128/AEM.00330-16

Wilson, Z.N., Scott, A.L., Dowell, R.D., and Odorizzi, G. (2018). PI(3,5)P_2 controls vacuole potassium transport to support cellular osmoregulation. *Mol. Biol. Cell* 29, 1718-1731. doi: 10.1091/mbc.E18-01-0015

Xu, H., Fang, T., Yan, H., and Jiang, L. (2019). The protein kinase Cmk2 negatively regulates the calcium/calcineurin signalling pathway and expression of calcium pump genes *PMR1* and *PMC1* in budding yeast. *Cell Commun. Signal.* 17, 7. doi: 10.1186/s12964-019-0320-z
